# Deep Reinforcement Learning Controller for 3D Path Following and Collision Avoidance by Autonomous Underwater Vehicles

**DOI:** 10.3389/frobt.2020.566037

**Published:** 2021-01-25

**Authors:** Simen Theie Havenstrøm, Adil Rasheed, Omer San

**Affiliations:** ^1^ Department of Engineering Cybernetics, Norwegian University of Science and Technology, Trondheim, Norway; ^2^ Mathematics and Cybernetics, SINTEF Digital, Trondheim, Norway; ^3^ School of Mechanical and Aerospace Engineering, Oklahoma State University, Stillwater, OK, United States

**Keywords:** continuous control, collision avoidance, path following, deep reinforcement learning, autonomous under water vehicle, curriculum learning

## Abstract

Control theory provides engineers with a multitude of tools to design controllers that manipulate the closed-loop behavior and stability of dynamical systems. These methods rely heavily on insights into the mathematical model governing the physical system. However, in complex systems, such as autonomous underwater vehicles performing the dual objective of path following and collision avoidance, decision making becomes nontrivial. We propose a solution using state-of-the-art Deep Reinforcement Learning (DRL) techniques to develop autonomous agents capable of achieving this hybrid objective without having a priori knowledge about the goal or the environment. Our results demonstrate the viability of DRL in path following and avoiding collisions towards achieving human-level decision making in autonomous vehicle systems within extreme obstacle configurations.

## 1 Introduction

Autonomous Underwater Vehicles (AUVs) are used in many subsea commercial applications such as seafloor mapping, inspection of pipelines and subsea structures, ocean exploration, environmental monitoring, and various research operations. The wide range of operational contexts implies that truly autonomous vehicles must be able to follow spatial trajectories (path following), avoid collisions along these trajectories (collision avoidance), and maintain a desired velocity profile (velocity control). In addition, AUVs are often underactuated by the fact that they operate with three generalized actuators (propeller, elevation, and rudder fins) in six degrees of freedom (6-DOF) (Fossen, 2011). The complexity that arises when combining the control objectives, a complicated hydrodynamic environment and disturbances, and the physical design with three generalized actuators spurs an intriguing control challenge. The current work is an attempt to address these challenges. Since path following and collision avoidance are the two main challenges addressed in this paper, a brief overview of the state of the art is provided in the following subsections.

### 1.1 Path Following

The path-planning and path-following problems are heavily researched and documented in classical control literature. The control objective is to plan a collision-free optimal path, defined relative to some inertial frame, and minimize tracking errors, i.e., the distance between the vehicle and the path. For road vehicles, several path/motion planning strategies are demonstrated (Karaman and Frazzoli, 2011). In their recent studies, Ljungqvist et al. (2019) and Cirillo (2017) demonstrated the use of the Lattice-based method for path/motion planning. In both works where the focus was path planning, the algorithms developed were tested on real vehicles. Pivtoraiko et al. (2009) contributed with a principled mechanism to construct an efficient, precise, and deferentially constrained search space upon which any planner might operate. However, most of these works were limited to fully actuated surface vehicles. Three-dimensional (3D) path following involves tracking errors that are composed of horizontal and vertical components and forms an accurate representation of the real engineering operations for AUVs (Chu and Zhu, 2015). Typically, a variant of the Proportional Integral Derivative (PID) controller based on reduced order models (ROM) is used to control elevator and rudder to eliminate tracking errors (Fossen, 2011, ch. 12).

More advanced approaches are also available. A classical nonlinear approach is found in Encarnacao and Pascoal (2000), where a kinematic controller was designed based on Lyapunov theory and integrator backstepping. To extend the nonlinear approach reliably in the presence of disturbances and parametric uncertainties, Chu and Zhu (2015) proposed an adaptive sliding mode controller, where an adaptive control law is implemented using a radial basis function neural network. To alleviate chattering, a well-known “zig-zag” phenomenon occurring when implementing sliding mode controllers due to a finite sampling time, an adaptation rate was selected based on the so-called minimum disturbance estimate. Xiang et al. (2017) proposed fuzzy logic for adaptive tuning of a feedback linearization PID controller. The heuristic, adaptive scheme accounts for modeling errors and time-varying disturbances. They also compare the performance on 3D path following with conventional PID and nonadaptive backstepping-based controllers, both tuned with inaccurate and accurate model parameters, to demonstrate the robust performance of the suggested controller. Liang et al. (2018) suggested using fuzzy backstepping sliding mode control to tackle the control problem. Here, the fuzzy logic was used to approximate terms for the nonlinear uncertainties and disturbances, specifically for use in the update laws for the controller design parameters.

Many other methods exist, but most published works on the 3D path-following problem incorporate either fuzzy logic, variants of PID control, backstepping techniques, or any combination thereof. More recently, there have been numerous attempts to achieve path following and motion control for AUVs by applying machine learning (ML) directly to low-level control. Specifically, Deep Reinforcement Learning (DRL) seems to be the favored approach. DRL controllers are based on experience gained from self-play or exploration, using algorithms that can learn to execute tasks by reinforcing good actions based on a performance metric. Yu et al. (2017) used a DRL algorithm known as Deep Deterministic Policy Gradients (DDPG) (Lillicrap et al., 2015) to obtain a controller that outperformed PID on trajectory tracking for AUVs. A DRL controller for underactuated marine vessels was implemented in Martinsen and Lekkas (2018a) to achieve path following for straight-line paths and later in Martinsen and Lekkas (2018b) for curved paths using transfer learning from the first study. The DRL controller demonstrated excellent performance, even compared to traditional Line-of-Sight (LOS) guidance. More recently, Meyer et al. (2020b) demonstrated that a DRL controller can achieve very impressive results in achieving the combined objective of path following and collision avoidance with a complex layout of stationary obstacles. Exciting results validating the real-world applications of DRL controllers for AUVs and unmanned surface vehicles can be found in Carlucho et al. (2018) and Woo et al. (2019). The first paper implemented the controller on an AUV equipped with six thrusters configured to generate actuation in pitch moment, yaw moment, and surge force. They demonstrated velocity control in both linear and angular velocities. The latter paper implemented a DRL controller on an unmanned surface vehicle with path following as the control objective and presented impressive experimental results from the full-scale test. Common to all these studies is that all the potential of DRL in path following is demonstrated in a 2D context only.

### 1.2 Collision Avoidance

Collision Avoidance (COLAV) system is an important part of the control systems for all types of autonomous vehicles. AUVs are costly to produce and typically equipped with expensive gears as well. Therefore, maximum efforts must be made to ensure their safe movements at all times. COLAV systems must be able to do *obstacle detection* using sensor data and information processing and *obstacle avoidance* by applying steering commands based on detection and avoidance logic. The two fundamental perspectives of COLAV control architectures are described in the literature: *deliberate* and *reactive* (Tan, 2006).

Deliberate architectures are plan driven and therefore necessitates a priori information about the environment and terrain. It could be integrated as part of the on-board guidance system (McGann et al., 2008), or at an even higher level in the control architecture, such as a waypoint planner (Ataei and Yousefi-Koma, 2015). Popular methods to solve the path-planning problem include A* algorithms (Carroll et al., 1992; Garau et al., 2005), genetic algorithms (Sugihara and Yuh, 1996), and probabilistic roadmaps (Kavraki et al., 1996; Cashmore et al., 2014). Deliberate methods are computationally expensive, due to information processing about the global environment. However, they are more likely to make the vehicle converge to the objective (Eriksen et al., 2016). Reactive methods on the other hand are faster and process only real-time sensor data to make decisions. In this sense, the reactive methods are considered local and are used when rapid action is required. Examples of reactive methods are the dynamic window approach (Fox et al., 1997; Eriksen et al., 2016), artificial potential fields (Williams et al., 1990), and constant avoidance angle (Wiig et al., 2018). A potential pitfall with reactive methods is local minima manifested as dead ends (Eriksen et al., 2016).

To improve both the deliberate and the reactive approach, a *hybrid* approach is used in practice by combining the strengths of both. Such architectures are comprised of deliberate, reactive, and execution layers. The deliberate layer handles high-level planning, while the reactive layer tackles incidents happening in real time. The execution layer facilitates the interaction between the deliberate and reactive architectures and decides the final commanded steering (Tan, 2006). The hybrid approach is demonstrated in Meyer et al. (2020a) where a DRL agent trained in a purely synthetic environment could achieve the combined objective of path following and collision avoidance with real sea traffic data (moving obstacles) in the Trondheim fjord while complying with collision avoidance regulations. There are still challenges in state-of-the-art COLAV methods for vehicles subjected to nonholonomic constraints, such as AUVs. Instability issues, neglecting vehicle dynamics and actuator constraints leading to infeasible reference paths, and algorithms causing the vehicle to stop are recurring challenges seen in the literature. Additionally, extensive research discusses methods for COLAV in 2D that cannot be directly applied to 3D. In many cases where such methods are adapted to 3D, however, they do not optimally take advantage of the extra dimension (Wiig et al., 2018).

### 1.3 Research Goals and Methods

The current study is aimed at the following:

• Figuring out if it is possible to tame an underactuated AUV with 6-DOF to achieve the combined objective of path following and collision avoidance in 3D using DRL. It is hoped that this will provide additional insight into the dynamical system from a new perspective.
• Exploring the potential of curriculum learning (Bengio et al., 2009) in the context of training AUVs. The basic idea behind curriculum learning is to start small, learn easier aspects of the task or easier subtasks, and then gradually increase the difficulty level.

In order to achieve the research goals, we employ a DRL controller as the motion control system operating the control fins of the AUV, to learn the control law through exploration. As the DRL algorithm, we have used the Policy Proximal Optimization (PPO), proposed by Schulman et al. (2017). The agent commands the control fins, while a traditional PI-controller maintains a desired cruise speed. The key idea lies in the fact that the agent learns to operate both the elevator and rudder at the same time and should therefore be able to learn an optimal strategy for navigating in both planes. The challenge of DRL control is establishing a reward function such that safe and effective tracking behavior is incentivized. To implement curriculum learning, scenarios ranging from *beginner* to *expert* level difficulty are constructed. Initially, the agent starts with only a path without any obstacle or ocean current disturbances and trains until it masters that difficulty level. Then, obstacles are progressively added and eventually ocean current disturbances are introduced to form the expert level scenario. The scenarios are detailed in Section 3.1. In a COLAV sense, the predefined path can be viewed as the deliberate architecture, where it is assumed that the waypoints are generated by some path-planning scheme, and the random and unforeseen obstacles are placed on this presumed collision-free path. The DRL agent operates in effect as the reactive system that must handle the threat of collisions rapidly but at the same time chooses effective trajectories to reach the target.

The article is organized as follows: In Section 2, the background theory on AUV modeling, the path-generation, and DRL is given. The implementation of the simulation model and the utilized DRL algorithm are briefly described in Section 3. Section 4 presents the results followed by conclusions and proposed future work in Section 5.

## 2 Theory

### 2.1 AUV Model

This section introduces a dynamic model that can be used to accurately simulate an AUV in a hydrodynamic environment. This is done by using a 6-DOF maneuvering model which is represented by 12 highly coupled and nonlinear first-order ordinary differential equations (ODEs). Dynamic models for AUVs comprise a *kinematic* (Section 2.1.2) and a *kinetic* (Section 2.1.3) part. Kinematics represent the geometrical evolution of the vehicle and involves a coordinate transformation between two important reference frames. Kinetics considers the forces and moments causing vehicle motion. The kinetic analysis is typically important when designing motion control systems because actuation can only be achieved by applying control forces and moments. Before delving into the details of the kinematic and kinetic equations, the notation used to detail the model’s states and parameters are presented in Table 1. This notation is used by the Society of Naval Architects and Marine Engineers (SNAME (1950)) (Fossen, 2011, p. 16).

**TABLE 1:** Notation for marine vessels as given by SNAME (1950).

Degree of freedom	Forces and moments	Velocities	Positions
1 Translation in the *x* direction (surge)	*T*	*u*	*x*
2 Translation in the *y* direction (sway)	*Y*	*v*	*y*
3 Translation in the *z* direction (heave)	*Z*	*w*	*z*
4 Rotation about *x* axis (roll)	*K*	*q*	ϕ
5 Rotation about *y* axis (pitch)	*M*	*p*	θ
6 Rotation about *z* axis (yaw)	*N*	*r*	ψ

#### 2.1.1 Reference Frames

Two reference frames are especially important in the modeling of vehicle dynamics. The North-East-Down (NED) frame denoted {n} and the body frame denoted {b}. The NED coordinate system is considered to be inertial, with principal axis pointing towards true north, east, and downwards—normal to Earth’s surface—for the $x_{n},y_{n},z_{n}$ axes, respectively. Since the NED frame is considered inertial, Newton’s laws of motion apply. The body frame has its origin located at the vehicle’s center of control (CO), which in general is a design choice. The CO is not automatically placed at the vehicle’s center of mass (CM) since this point might be time-varying. A typical point for the CO for AUVs is therefore the center of buoyancy (CB). The body frame’s $x_{b}$ axis points along the longitudinal axis of the vehicle, the $y_{b}$ axis points transversal, and the $z_{b}$ axis points normal to the vehicle surface. To relate vectors in different coordinates, we utilize the Euler-angle rotation matrix seen in Eq. 1.

$$R_{b}^{n}\left(\Theta_{nb}\right)=\left[\begin{array}{ccc} c\psi c\theta & -s\psi c\phi +c\psi s\theta s\phi & s\psi s\phi +c\psi c\phi s\theta \\ s\psi c\theta & c\psi c\phi +s\phi s\theta s\psi & -c\psi s\phi +s\theta s\psi c\phi \\ -s\theta & c\theta s\phi & c\theta c\phi \end{array}\right],$$ (1)

where sϕ=sin⁡ϕ,cϕ=cos⁡ϕ.

The Euler angles describing the vehicle’s attitude is contained in $\Theta_{nb}={\left[\phi ,\theta ,\psi \right]}^{T}$. To obtain a vector expressed in the body frame in NED coordinates, a matrix multiplication with the rotation matrix is applied. To rotate the inverse way, i.e., from {n} to {b}, we use the transposed rotation matrix ${\left(R_{b}^{n}\right)}^{T}=R_{n}^{b}$.

#### 2.1.2 Kinematic Equations

The kinematic state vector is the concatenation of the position of the vehicle in NED coordinates and the vehicle’s attitude with respect to the NED frame. This vector is symbolized by $\eta ={\left[p^{n},\Theta_{nb}\right]}^{T}={\left[x,y,z,\phi ,\theta ,\psi \right]}^{T}$. The velocity vector expressed in {b}, $v^{b}$, is utilized to find a differential equation for $p^{n}$. Rotating this vector by applying Eq. 1 yields the differential equation for the position in {n}:

$${\dot{p}}^{n}=v^{n}=R_{b}^{n}\left(\Theta_{nb}\right)v^{b},$$ (2)

where the body-fixed velocity vector is defined as $v^{b}={\left[u,v,w\right]}^{T}$ and the components are defined according to Table 1.

To write a differential equation for the whole kinematic state vector, an equation describing the time-evolution of the Euler angles is obtained by transforming the linear velocities expressed in {b}, according to Eq. 3. Note that this transformation is not well defined for θ=(π/2). An alternative representation avoiding the singularity is quaternion parameterization (Fossen, 2011, p. 25).

$${\dot{\Theta }}_{nb}=T_{\text{Θ}}\left(\Theta_{nb}\right)\omega_{b/n}^{b}=\left[\begin{array}{ccc} 1 & s\phi t\theta & c\phi t\theta \\ 0 & c\phi & -s\phi \\ 0 & \frac{s\phi }{c\theta } & \frac{c\phi }{c\theta } \end{array}\right]\left[\begin{array}{c} q\\ p\\ r \end{array}\right]$$ (3)

where tθ=tanθ. Combining Eqs. 2, 3, the complete kinematic differential equation can be written as Eq. 4.

$$\dot{\eta }=\left[\begin{array}{c} {\dot{p}}^{n}\\ {\dot{\Theta }}_{nb} \end{array}\right]=\left[\begin{array}{cc} R_{b}^{n}\left(\Theta_{nb}\right) & 0\\ 0 & T_{\text{Θ}}\left(\Theta_{nb}\right) \end{array}\right]\left[\begin{array}{c} v^{b}\\ \omega_{b/n}^{b} \end{array}\right]=J_{\text{Θ}}\left(\eta \right)\nu .$$ (4)

#### 2.1.3 Kinetic Equations

The kinetic equations of motion for a marine craft can be expressed as a mass-spring-damper system. The mass terms naturally stem from vessel body, while the spring forces acting on the body arise from buoyancy. The damping is a result of the hydrodynamic forces caused by motion. The model implemented is adapted from da Silva et al. (2007) and all model parameters can be seen in Table 2. The AUV specifications on which the model parameters are based are given by the tables given in the appendix. Furthermore, it is based on the following assumptions:

• The AUV operates at a depth below disturbances from wind and waves.
• The maximum speed is 2m/s.
• The moment of inertia can be approximated by that of a spheroid.
• The AUV is passively stabilized in roll and pitch by placing the CM a distance $z_{G}$ under the CO.
• The AUV shape is top-bottom and port-starboard symmetric.
• As a fail-safe mechanism, the AUV is slightly buoyant.

**TABLE 2:** AUV model parameters.

Parameter	Description	Value
Mass and Coriolis matrix		
*m*	Mass	18
$Z_{g}$	COG relative to CO	0.01
$I_{x}$	Moment of inertia, roll	0.0405
$I_{y}$	Moment of inertia, pitch	1.070
$I_{z}$	Moment of inertia, yaw	1.070
$X_{\dot{u}}$	Added mass, surge	−1.029
$Y_{\dot{v}}$	Added mass, sway	−16.153
$Z_{\dot{w}}$	Added mass, heave	−16.153
$K_{\dot{p}}$	Added mass, roll	0
$M_{\dot{q}}$	Added mass, pitch	−0.758
$N_{\dot{r}}$	Added mass, yaw	−0.758
Damping matrix		
$X_{u}$	Linear damping, surge	−2.4
$Y_{v}$	Linear damping, sway	−23
$Z_{w}$	Linear damping, heave	−23
$K_{p}$	Linear damping, roll	−0.3
$M_{q}$	Linear damping, pitch	−9.7
$N_{r}$	Linear damping, yaw	−9.7
$Y_{r}$	Linear damping, yaw on sway	11.5
$Z_{q}$	Linear damping, pitch on heave	−11.5
$M_{w}$	Linear damping, heave on pitch	3.1
$N_{v}$	Linear damping, sway on yaw	−3.1
$X_{u\left|u\right|}$	Nonlinear damping, surge	−2.4
$Y_{v\left|v\right|}$	Nonlinear damping, sway	−80
$Z_{w\left|w\right|}$	Nonlinear damping, heave	−80
$K_{p\left|p\right|}$	Nonlinear damping, roll	−6.4e-4
$M_{q\left|q\right|}$	Nonlinear damping, pitch	−9.1
$N_{r\left|r\right|}$	Nonlinear damping, yaw	−9.1
$Y_{r\left|r\right|}$	Nonlinear damping, yaw on sway	0.3
$Z_{q\left|q\right|}$	Nonlinear damping, pitch on heave	−0.3
$M_{w\left|w\right|}$	Nonlinear damping, heave on pitch	1.5
$N_{v\left|v\right|}$	Nonlinear damping, sway on yaw	−1.5
$Y_{uv_{f}}$	Fin lift, sway	−19.2
$Z_{uw_{f}}$	Fin lift, heave	−19.2
$M_{uq_{f}}$	Fin lift, pitch	−3.072
$N_{uq_{f}}$	Fin lift, yaw	−3.072
$Y_{ur_{f}}$	Fin lift, yaw on sway	7.68
$Z_{uq_{f}}$	Fin lift, pitch on heave	−7.68
$M_{uw_{f}}$	Fin lift, heave on pitch	−7.68
$N_{uv_{f}}$	Fin lift, sway on yaw	7.68
$Y_{uv_{b}}$	Body lift, sway	−10.956
$Z_{uw_{b}}$	Body lift, heave	−10.956
$M_{uw_{b}}$	Body lift, heave on pitch	−3.309
$N_{uv_{b}}$	Body lift, sway on yaw	3.309
Restoring force matrix		
*W*	Weight	176.58
*B*	Buoyancy	177.58
Control force matrix		
$Y_{uu\delta_{r}}$	Rudder fin on sway	19.2
$N_{uu\delta_{r}}$	Rudder fin on yaw	7.68
$Z_{uu\delta_{s}}$	Elevator fin on heave	−19.2
$M_{uu\delta_{s}}$	Elevator fin on pitch	−7.68

The vessel’s motion is governed by the nonlinear kinetic equations expressed in {b} according to Equation 5:

$$\underset{\text{Mass}\;\text{forces}}{\underset{︸}{M{\dot{\nu }}_{r}}}+\underset{\text{Coriolis}\;\text{forces}}{\underset{︸}{C\left(\nu_{r}\right)\nu_{r}}}+\underset{\text{Damping}\;\text{forces}}{\underset{︸}{D\left(\nu_{r}\right)\nu_{r}}}+\underset{\text{Restoring}\;\text{forces}}{\underset{︸}{g\left(\eta \right)}}=\tau_{\text{control}},$$ (5)

where $\nu_{r}=\nu -\nu_{c}$ is the velocity relative to the velocity of an ocean current, represented by $\nu_{c}$ in {b} . When no currents are present, we see that $\nu =\nu_{r}$. Furthermore, only irrotational currents are considered.

##### 2.1.3.1 *Mass Forces*

The systems inertia matrix, M, is the sum of the inertia matrix for the rigid body (RB) and the added mass (A). Added mass is the inertia added from the weight of the fluid that the vessel displaces when moving through it. Because of the symmetry assumptions, both matrices are diagonal. However, the rigid body matrix is defined in the center of gravity, such that it must be shifted to the center of control, yielding some coupling terms:

$$M=M_{RB}+M_{A}=\left[\begin{array}{cccccc} m-X_{\dot{u}} & 0 & 0 & 0 & mz_{G} & 0\\ 0 & m-Y_{\dot{v}} & 0 & -mz_{G} & 0 & 0\\ 0 & 0 & m-Z_{\dot{w}} & 0 & 0 & 0\\ 0 & -mz_{G} & 0 & I_{x}-K_{\dot{p}} & 0 & 0\\ mz_{G} & 0 & 0 & 0 & I_{y}-M_{\dot{q}} & 0\\ 0 & 0 & 0 & 0 & 0 & I_{z}-N_{\dot{r}} \end{array}\right].$$ (6)

##### 2.1.3.2 *Coriolis Forces*

Naturally, the added mass will also affect the Coriolis-centripetal matrix, $C\left(\nu_{r}\right)$, which defines the forces occurring due to {b} rotating about {n}. Moreover, the linear-velocity independent parameterization of the rigid body Coriolis-centripetal matrix is utilized, easing the implementation of irrotational ocean currents (Fossen, 2011, p. 222) (note that there are still linear-velocity terms caused by the added mass). It is this trick that makes it possible to collect the rigid body and add mass terms to represent the 6-DOF model by the elegant Eq. 5. When using the linear-velocity independent parameterization, the Coriolis-centripetal matrix is written as

$$C\left(\nu_{r}\right)=C{\left(\nu_{r}\right)}_{RB}+C{\left(\nu_{r}\right)}_{A}=\left[\begin{array}{cccccc} 0 & -mr & mq & mz_{G}r & -Z_{\dot{w}}w_{r} & Y_{\dot{v}}v_{r}\\ mr & 0 & -mp & Z_{\dot{w}}w_{r} & mz_{G}r & -X_{\dot{u}}u_{r}\\ -mq & mp & 0 & -\left(mz_{G}p+Y_{\dot{v}}v_{r}\right) & -mz_{G}q+X_{\dot{u}}u_{r} & 0\\ -mz_{G}r & -Z_{\dot{w}}w_{r} & mz_{G}p+Y_{\dot{v}}v_{r} & 0 & \left(I_{z}-mz_{G}^{2}-N_{\dot{r}}\right)r & \left(-I_{y}+M_{\dot{q}}\right)q\\ Z_{\dot{w}}w_{r} & -mz_{G}r & mz_{G}q-X_{\dot{u}}u_{r} & (-I_{z}+mz_{G}^{2}+N_{\dot{r}})r & 0 & (I_{x}-K_{\dot{p}}p\\ -Y_{\dot{v}}v_{r} & X_{\dot{u}}u_{r} & 0 & (I_{y}-M_{\dot{q}})q & \left(-I_{x}+K_{\dot{p}}\right)p & 0 \end{array}\right].$$ (7)

##### 2.1.3.3 *Damping Forces*

The components of hydrodynamic damping modeled are linear viscous damping, nonlinear (quadratic) damping due to vortex shedding, and lift forces from the body and control fins. Thus, the damping matrix, $D\left(\nu_{r}\right)$, can be expressed as

$$D\left(\nu_{r}\right)=D+D_{n}\left(\nu_{r}\right)+L\left(\nu_{r}\right).$$ (8)

The linear damping is given by

$$D=-\left[\begin{array}{cccccc} X_{u} & 0 & 0 & 0 & 0 & 0\\ 0 & Y_{v} & 0 & 0 & 0 & Y_{r}\\ 0 & 0 & Z_{w} & 0 & Z_{q} & 0\\ 0 & 0 & 0 & K_{p} & 0 & 0\\ 0 & 0 & M_{w} & 0 & M_{q} & 0\\ 0 & N_{v} & 0 & 0 & 0 & N_{r} \end{array}\right].$$

The nonlinear damping is given by

$$D_{n}\left(\nu \right)=-\left[\begin{array}{cccccc} X_{u|u|}|u| & 0 & 0 & 0 & 0 & 0\\ 0 & X_{v|v|}\left|v\right| & 0 & 0 & 0 & Y_{r|r|}\left|r\right|\\ 0 & 0 & Z_{w|w|}\left|w\right| & 0 & Z_{q|q|}\left|q\right| & 0\\ 0 & 0 & 0 & K_{p|p|}\left|p\right| & 0 & 0\\ 0 & 0 & M_{w|w|}\left|w\right| & 0 & M_{q|q|}\left|q\right| & 0\\ 0 & N_{v|v|}\left|v\right| & 0 & 0 & 0 & N_{r|r|}\left|r\right| \end{array}\right].$$

Finally, the lift is given by

$$L\left(\nu \right)=-\left[\begin{array}{cccccc} 0 & 0 & 0 & 0 & 0 & 0\\ 0 & Y_{uv_{f}}+Y_{uv_{b}} & 0 & 0 & 0 & Y_{ur_{f}}\\ 0 & 0 & Z_{uw_{f}}+Z_{uw_{b}} & 0 & Z_{uq_{f}} & 0\\ 0 & 0 & 0 & 0 & 0 & 0\\ 0 & 0 & M_{uw_{f}}+M_{uw_{b}} & 0 & M_{uq_{f}} & 0\\ 0 & N_{uv_{f}}+N_{uv_{b}} & 0 & 0 & 0 & N_{ur_{f}} \end{array}\right]u.$$

##### 2.1.3.4 *Restoring Forces*

The restoring forces working on the AUV body are functions of the orientation, weight, and buoyancy of the vehicle. Because the vehicle is assumed to be slightly buoyant and the passive stabilization of roll and pitch, the restoring force vector can be written as

$$G\left(\eta \right)=\left[\begin{array}{c} \left(W-B\right)\sin\theta \\ -\left(W-B\right)\cos\theta \sin\phi \\ -\left(W-B\right)\cos\theta \cos\phi \\ z_{G}W\cos\theta \sin\phi \\ z_{G}W\sin\theta \\ 0 \end{array}\right].$$ (9)

##### 2.1.3.5 *Control Inputs*

There are three control inputs: propeller thrust, rudder, and elevator fins denoted as $n,\delta_{r}$ and $\delta_{s}$, respectively. All actuators are constrained according to Table 3. The constraint on the thrust force guarantees that the low-speed assumption holds. The control inputs are related to the control force vector according to Eq. 10:

$$\tau_{\text{control}}=\left[\begin{array}{ccc} 1 & 0 & 0\\ 0 & Y_{uu\delta_{r}}u_{r}^{2} & 0\\ 0 & 0 & Z_{uu\delta_{s}}u_{r}^{2}\\ 0 & 0 & 0\\ 0 & 0 & M_{uu\delta_{s}}u_{r}^{2}\\ 0 & N_{uu\delta_{r}}u_{r}^{2} & 0 \end{array}\right]\left[\begin{array}{c} n\\ \delta_{r}\\ \delta_{s} \end{array}\right].$$ (10)

**TABLE 3:** Specifications for simulated AUV adapted from da Silva et al. (2007).

Symbol	Description	Value	Unit
*m*	Mass	18	kg
*L*	Length	108	cm
*W*	Weight	176	N
*B*	Buoyancy	177	N
$z_{G}$	Position of CM w.r.t. CB in *z* axis	1	cm
*d*	Diameter	15	cm
$\delta_{\max}$	Maximum control fin deflection	$30^{\circ }$	deg
$\eta_{\max}$	Maximum propeller thrust	14	N

This completes the details of the model implemented. The numerical values used in the simulation can be found in Table 2. For a complete derivation of the model and how the numerical values are obtained from the specifications and assumptions, da Silva et al. (2007) and Fossen (2011) are referred to for extensive explanations.

#### 2.1.4 Simulation Model for Ocean Current

To simulate the environmental disturbances in the form of ocean currents, a 3D irrotational ocean current model is implemented. The model is based on generating the intensity of the current, $V_{c}={\left\|\nu_{c}\right\|}_{2}$, by utilizing a first-order *Gauss-Markov Process* (Fossen, 2011, Ch. 8):

$${\dot{V}}_{c}=-\mu V_{c}+w,$$ (11)

where *w* is *white noise* and μ≥0 a constant. An integration limit is set so that the current speed is limited between 0.5 to 1 m/s. The current direction is static and initialized randomly for each episode. The current direction is described by the sideslip angle and angles of attack are symbolized by $\alpha_{c}$ and $\beta_{c}$, respectively. These angles represent from what direction the current hits the body frame. In NED coordinates, the linear ocean current velocities can be obtained by Eq. 12 (Fossen, 2011, Ch. 8).

$$v_{c}^{n}=V_{c}\left[\begin{array}{c} \cos\alpha_{c}\cos\beta_{c}\\ \sin\beta_{c}\\ \sin\alpha_{c}\cos\beta_{c} \end{array}\right].$$ (12)

There are no dynamics associated with the sideslip angle and the angle of attack in the simulations. The current direction stays fixed throughout an episode. To obtain the linear velocities in the body frame, we apply the inverse Euler-angle rotation matrix, as seen in Eq. 13:

$$\left[\begin{array}{c} u_{c}\\ v_{c}\\ w_{c} \end{array}\right]=R_{b}^{n}{\left(\Theta_{nb}\right)}^{T}v_{c}^{n}.$$ (13)

Since the ocean current is defined to be irrotational, the full current velocity vector is written $\nu_{c}=\left[u_{c},v_{c},w_{c},0,0,0\right]$.

#### 2.1.5 Control Fin Dynamics

To simulate the operation of the control fins more realistically, the output of the controller passes through a first-order low-pass filter with time constant $T_{f}$. The intention behind this implementation is to remove noisy outputs from the DRL agent, without having to add a cost to the control action derivatives ${\dot{\delta }}_{r}$ and ${\dot{\delta }}_{s}$. The implementation of the discrete low-pass filter for the control fins is given by Equation 14:

$$\delta_{i,t}=\left(1-a\right)\delta_{i,t-1}+au_{t},i=r\;\text{or}\;s$$ (14)

where the filter parameter *a* is related to the time constant by $a=\text{Δ}t/\left(T_{f}+\text{Δ}t\right)$, $u_{t}$ is the raw control action, and Δt is the simulation step size (Haugen, 2008).

### 2.2 3D Path Following

In this section, the path-following problem is formally introduced. A set of $n_{w}$ waypoints is used to generate the path, starting at the origin of the NED coordinates for simplicity. Any well-defined path for a vehicle that cannot accelerate infinitely fast must be $G^{2}$ continuous. Methods such as cubic and spline interpolation establish $G^{2}$ continuity and are straightforward to implement in 2D but cannot be applied directly in 3D interpolation. In fact, some spline methods have been shown to produce paths that do not visit all waypoints in 3D (Chang and Huh, 2015). This is undesirable as the path should visit all waypoints in the correct sequence. Chang and Huh (2015) proposed a 3D extension of quadratic polynomial interpolation (QPMI) to create a $G^{2}$ continuous path by using second-order polynomials and a membership function to smoothly switch between polynomials. They choose quadratic polynomials because this is the lowest order possible for obtaining $G^{2}$ continuity, and higher-order polynomials are prone to be corrupted by Runge’s phenomenon. To generate a QPMI path in 3D, we start by writing the path $P_{p}$ as a function of the along-track distance, *s*, such that $P_{p}\left(s\right):\left(x\left(s\right),y\left(s\right),z\left(s\right)\right)$. Each waypoint *m* has a Euclidian distance $s_{m}$ associated with it. For the first waypoint, this distance is zero, i.e., $s_{1}=0$, and the others are obtained by the generalized equation $s_{m}=\sum_{i=2}^{m}\sqrt{{\left(x_{i}-x_{i-1}\right)}^{2}+{\left(y_{i}-y_{i-1}\right)}^{2}+{\left(z_{i}-z_{i-1}\right)}^{2}}$. The quadratic polynomials linking three waypoints together can be written as

$$\begin{array}{c} x_{m}\left(s\right)=a_{x_{m}}s^{2}+b_{x_{m}}s+c_{x_{m}},\\ y_{m}\left(s\right)=a_{y_{m}}s^{2}+b_{y_{m}}s+c_{y_{m}},\\ z_{m}\left(s\right)=a_{z_{m}}s^{2}+b_{z_{m}}s+c_{z_{m}},\\ m=2,3,\ldots ,n_{w}-1. \end{array}$$ (15)

And the coefficients can be found by solving the following matrix equations:

$$\left[\begin{array}{c} a_{x_{m}}\\ b_{x_{m}}\\ c_{x_{m}} \end{array}\right]={\left[\begin{array}{ccc} s_{m-1}^{2} & s_{m-1} & 1\\ s_{m}^{2} & s_{m} & 1\\ s_{m+1}^{2} & s_{m+1} & 1 \end{array}\right]}^{-1}\left[\begin{array}{c} x\left(s_{m-1}\right)\\ x\left(s_{m}\right)\\ x\left(s_{m+1}\right) \end{array}\right],$$ (16)

$$\left[\begin{array}{c} a_{y_{m}}\\ b_{y_{m}}\\ c_{y_{m}} \end{array}\right]={\left[\begin{array}{ccc} s_{m-1}^{2} & s_{m-1} & 1\\ s_{m}^{2} & s_{m} & 1\\ s_{m+1}^{2} & s_{m+1} & 1 \end{array}\right]}^{-1}\left[\begin{array}{c} y\left(s_{m-1}\right)\\ y\left(s_{m}\right)\\ y\left(s_{m+1}\right) \end{array}\right],$$ (17)

$$\begin{array}{c} \left[\begin{array}{c} a_{z_{m}}\\ b_{z_{m}}\\ c_{z_{m}} \end{array}\right]={\left[\begin{array}{ccc} s_{m-1}^{2} & s_{m-1} & 1\\ s_{m}^{2} & s_{m} & 1\\ s_{m+1}^{2} & s_{m+1} & 1 \end{array}\right]}^{-1}\left[\begin{array}{c} z\left(s_{m-1}\right)\\ z\left(s_{m}\right)\\ z\left(s_{m+1}\right) \end{array}\right],\\ m=2,3,\ldots ,n_{w}-1. \end{array}$$ (18)

A path represented by $n_{w}$ waypoints requires $n_{w}-2$ polynomials to generate the QPMI path, as seen in the previous equations. A group of polynomials linking three and three waypoints is therefore obtained. The group of polynomials representing the path is written as$P_{p}\left(s\right):\left(X\left(s\right),Y\left(s\right),Z\left(s\right)\right)$, where the group X(s),Y(s),Z(s) is expressed in a general form as

$$X\left(s\right)=\{\begin{array}{cc} x_{2}\left(s\right), & s_{1}\leq s<s_{2},\\ \mu_{r,m}\left(s\right)x_{m+1}\left(s\right)+\mu_{f,m}\left(s\right)x_{m}\left(s\right),\left(2\leq m<n_{w}-1\right), & s_{2}\leq s<s_{n_{w}-1},\\ x_{n_{w}-1}\left(s\right), & s_{n_{w}-1}\leq s\leq s_{n_{w}}, \end{array}$$ (19)

$$Y\left(s\right)=\{\begin{array}{cc} y_{2}\left(s\right), & s_{1}\leq s<s_{2},\\ \mu_{r,m}\left(s\right)y_{m+1}\left(s\right)+\mu_{f,m}\left(s\right)y_{m}\left(s\right),\left(2\leq m<n_{w}-1\right), & s_{2}\leq s<s_{n_{w}-1},\\ y_{n_{w}-1}\left(s\right), & s_{n_{w}-1}\leq s\leq s_{n_{w}}, \end{array}$$ (20)

$$Z\left(s\right)=\{\begin{array}{cc} z_{2}\left(s\right), & s_{1}\leq s<s_{2},\\ \mu_{r,m}\left(s\right)z_{m+1}\left(s\right)+\mu_{f,m}\left(s\right)z_{m}\left(s\right),\left(2\leq m<n_{w}-1\right) & s_{2}\leq s<s_{n_{w}-1},\\ z_{n_{w}-1}\left(s\right), & s_{n_{w}-1}\leq s\leq s_{n_{w}}, \end{array}$$ (21)

and $\mu_{r,m}\left(s\right),\mu_{f,m}\left(s\right)$ are membership functions given by

$$\begin{array}{l} \mu_{r,m}\left(s\right)=\frac{s-s_{m}}{s_{m+1}-s_{m}},\\ \mu_{f,m}\left(s\right)=\frac{s_{m+1}-s}{s_{m+1}-s_{m}},\\ m=2,3,\ldots ,n_{w}-1. \end{array}$$ (22)

Note that the first and the last polynomial are not overlapping any of the others; hence, the membership functions can be thought of as equal to one and zero in these regions. In the intermediate waypoints, the polynomials are blended smoothly by linearly increasing and decreasing the contribution of the two polynomials. In the paper by Chang and Huh (2015), they go on to prove $G^{2}$ continuity and details of the derivation of the method.

#### 2.2.1 Guidance Laws for 3D Path Following

To define the tracking errors, the Serret-Frenet ({SF}) reference frame associated with each point of the path is introduced. The $x_{SF}$ axis points tangent to the path, the $y_{SF}$ axis points normal to the path, and the $z_{SF}$ axis points an orthogonal direction to both such that $z_{SF}=x_{SF}\times y_{SF}$ (Encarnacao and Pascoal, 2000). The tracking-error vector, $\varepsilon ={\left[\bar{s},e,h\right]}^{T}$, is defined by the along-track, cross-track, and vertical-track error. The tracking-error vector points towards the closest point on the path from the vessel. Because the origin of the {SF} frame can be arbitrarily placed, the point on the path closest to the vessel is chosen as the origin in the simulation. This yields $\bar{s}=0$, which intuitively makes sense in a path-following scenario since the path is not dependent on time. There is therefore no error in the along-track distance component.

To achieve path following, it is important to align the velocity vector of the vessel in *n*, $V^{n}$, with the tangent vector of the path. When this is not the case, a user-specified look-ahead distance Δ is used to guide the vessel back to the path. Therefore, the desired course is not directed towards the closest point on the path. Rather it converges smoothly while the vessel returns back to the path further downstream. This is done by defining guidance laws for the desired pitch and heading which is based on the components of ε and Δ. First, we obtain the tracking errors by Equation 23 (Breivik and Fossen, 2009):

$$\varepsilon =R_{n}^{SF}{\left(\upsilon_{p},\chi_{p}\right)}^{T}\left(P^{n}-P_{p}^{n}\right),$$ (23)

where $P^{n}$ is the position of the vessel and $P_{p}^{n}$ is the closest point on the path. Now the desired azimuth and elevation angle can be calculated according to

$$\begin{array}{l} \chi_{d}\left(e\right)=\chi_{p}+\chi_{r}\left(e\right),\\ \upsilon_{d}\left(h\right)=\upsilon_{p}+\upsilon_{r}\left(h\right), \end{array}$$ (24)

where $\chi_{r}\left(e\right)=\text{arctan}\left(e/\text{Δ}\right)$ and $\upsilon_{r}\left(h\right)=\text{arctan}\left(h/\sqrt{e^{2}+{\text{Δ}}^{2}}\right)$. It is seen that driving *e* and *h* to zero will in turn drive the correction angles $\chi_{r}\left(e\right)$ and $\upsilon_{r}\left(h\right)$ to zero, and the velocity vector then aligns with the tangent of the path given when $\chi =\chi_{d}=\chi_{p}$ and $\upsilon =\upsilon_{d}=\upsilon_{p}$.

### 2.3 Deep Reinforcement Learning

In RL, an algorithm, an **agent**, makes an **observation** $s_{t}$ of an **environment** and performs an **action** $a_{t}$. The observation is referred to as the state of the system and is drawn from the state space S. The action is restricted to the well-defined action space A. When an RL task is not infinitely long but ends at some time *T*, we say that the problem is episodic and that each iteration through the task is an episode.

After performing an action, the agent receives a scalar **reward** signal $r_{t}=r\left(s_{t},a_{t}\right)$. The reward quantifies how good it was to choose action $a_{t}$ when being in state $s_{t}$. The objective of the agent is typical to maximize the expected cumulative reward. The action choices of the agent are guided by a **policy** π(s), which can be either deterministic or stochastic. In the case that the learning algorithm involves a neural network, the policy is parametrized by the learnable parameters of the network, denoted by θ. When the policy is stochastic and dependent on a neural network, we write $\pi \left(s\right)=\pi_{\theta }\left(a|s\right)$.

#### 2.3.1 Proximal Policy Optimization

The actor-critic algorithm known as Proximal Policy Optimization was proposed by Schulman et al. (2017). We briefly present the general theory and the algorithm which is used in this research. Let the value function V(s) represent the expected cumulative reward during an episode when following the current policy. In addition, let the state-action value function Q(a,s) define the expected cumulative reward by following the policy and by taking initial action *a*. Then the advantage function A(s,a) is given by

A(s,a)=Q(s,a)−V(s). (25)

The advantage function represents the difference in expected return by taking action *a* in state *s*, as opposed to the following policy. Because both Q(s,a) and V(s) are unknown, an estimate of the advantage function, ${\hat{A}}_{t}$, is calculated based on an estimate of the value function $\hat{V}\left(s\right)$, which is made by the critic neural network. When the value function is estimated, an alternative for estimating the advantage function is the generalized advantage estimate (GAE), given in Equation 26 Schulman et al. (2015).

$${\hat{A}}_{t}=\delta_{t}+\left(\gamma \lambda \right)\delta_{t+1}+\cdots +{\left(\gamma \lambda \right)}^{T-t+1}\delta_{T-1}\;\text{where }\delta_{t}=r_{t}+\gamma \hat{V}\left(s_{t+1}\right)-\hat{V}\left(s_{t}\right)$$ (26)

Here, *T* is a truncation point which is typically much smaller than the duration of an entire episode. As before, γ is the discount factor. As the GAE is a sum of uncertain terms, the tunable parameter 0≤λ≤1 is introduced to reduce variance. However, λ<1 makes the GAE biased towards the earlier estimates of the advantage function. Hence, choosing λ is a bias-variance trade-off.

The second key component in PPO is introducing a surrogate objective. It is hard to apply gradient ascent directly to the RL objective. Therefore, Schulman et al. suggest a surrogate objective such that an increase in the surrogate provably leads to an increase in the original objective Schulman et al. (2017). The proposed surrogate objective function is given by Equation 27.

$$L^{\text{CLIP}}\left(\theta \right)={\hat{E}}_{t}\left[\text{min}\left(\frac{\pi_{\theta }\left(a_{t}|s_{t}\right)}{\pi_{\theta_{\text{old}}}\left(a_{t}|s_{t}\right)}{\hat{A}}_{t},\text{clip}\left(\frac{\pi_{\theta }\left(a_{t}|s_{t}\right)}{\pi_{\theta_{old}}\left(a_{t}|s_{t}\right)},1-\epsilon ,1+\epsilon \right){\hat{A}}_{t}\right)\right].$$ (27)

The tuning parameter ϵ reduces the incentive to make very large changes to the policy at every step of the gradient ascent. This is necessary as the surrogate objective only estimates the original objective locally in a so-called trust region. During a training iteration, *N* actors (parallelized agents) are enabled to execute the policy and in that way sample trajectories for *T* timesteps. Then the GAE is computed based on the sampled trajectories, and the advantage estimation is used to optimize the surrogate objective for K epochs using minibatches of size M per update. The PPO method is seen in its general form in Algorithm 1 (Schulman et al., 2017).

Algorithm 1: Proximal Policy Optimization, actor-critic style

**for** iteration: 1,2... **do**
  **for** actor: 1,2...*N* **do**
   Run policy $\pi \theta_{\text{old}}$ for T time-steps
   Compute advantage estimate ${\hat{A}}_{1}\ldots {\hat{A}}_{T}$
  **end**
  Optimize surrogate L w.r.t. θ, with K epochs and mini-batch size *M* < *NT*
  $\theta_{\text{old}}\leftarrow \theta$
 **end**

## 3 Method and Implementation

The simulation environments are built to comply with the **OpenAI Gym** (Brockman et al., 2016) standard interface. For the RL algorithms, the **Stable Baselines** package which offers improved parallelizable implementations based on **OpenAI Baselines** (Dhariwal et al., 2017) library is utilized. The complete code can be found on Github^1^. Ten different scenarios are created: five for training and five for testing.

### 3.1 Environment Scenarios

Training scenarios are constructed by generating a path from a random set of $n_{w}$ waypoints which are generated such that unrealistically sharp turns are avoided. The first scenario used in curriculum learning is called *beginner*, where only a path and no obstacles or ocean current is present. Then the agent is introduced to the *intermediate* level, where a single obstacle is placed on the half-way mark. The next level is called *proficient*. Here, two more obstacles are placed equally distanced from the half-way mark.

**Figure 1:**
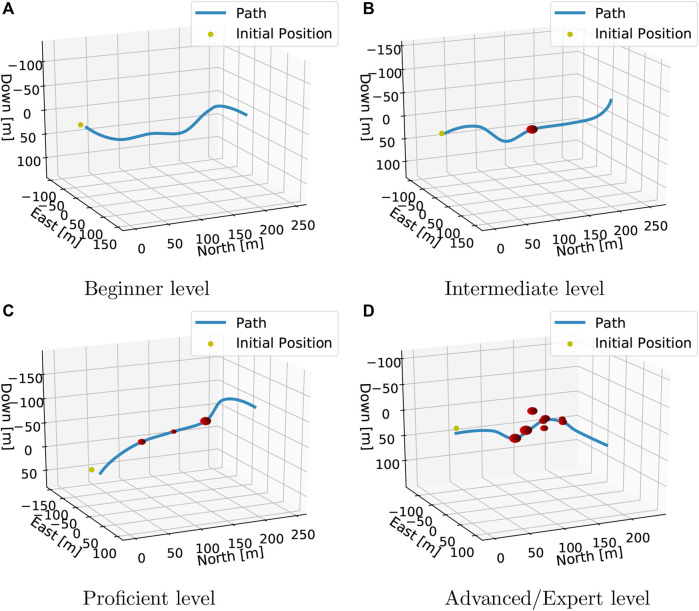
Training scenarios used in curriculum learning.

The last part of training happens in the *advanced* and *expert* level scenarios. In the advanced level difficulty, we generate the *proficient* challenge, but additionally five more obstacles are placed randomly off-path, such that an avoidance maneuver could induce a new collision course. The distinction between the expert and the advanced level is the inclusion of the ocean current disturbance. In all scenarios, the first and the last third of the path are collision-free, in order to keep part of the curriculum from the beginner scenario (pure path following) present throughout the learning process. This enables the agent to not forget knowledge learned from doing path following only. Figure 1 illustrates the different training contexts the agent is exposed to. In addition to training, the agent progressively through these scenarios, quantitative evaluation is performed by sampling a number of episodes such that the agents’ performance across the various difficulty levels can be established. After evaluating the controllers by statistical averages, qualitative analysis is done in designated test scenarios. These are designed to test specific aspects of the agents’ behavior. The first scenario tests a pure path following on a nonrandom path (in order for results to be reproducible) both with and without the presence of an ocean current. Next, special (extreme) cases where it would be preferable to use only one actuator for COLAV, i.e., horizontally and vertically stacked obstacles, are generated. The agents are also tested in a typical pitfall scenario for reactive COLAV algorithms: a dead end. See Section 4.2 for illustrations of the test scenarios.

### 3.2 Obstacle Detection

**Figure 2:**
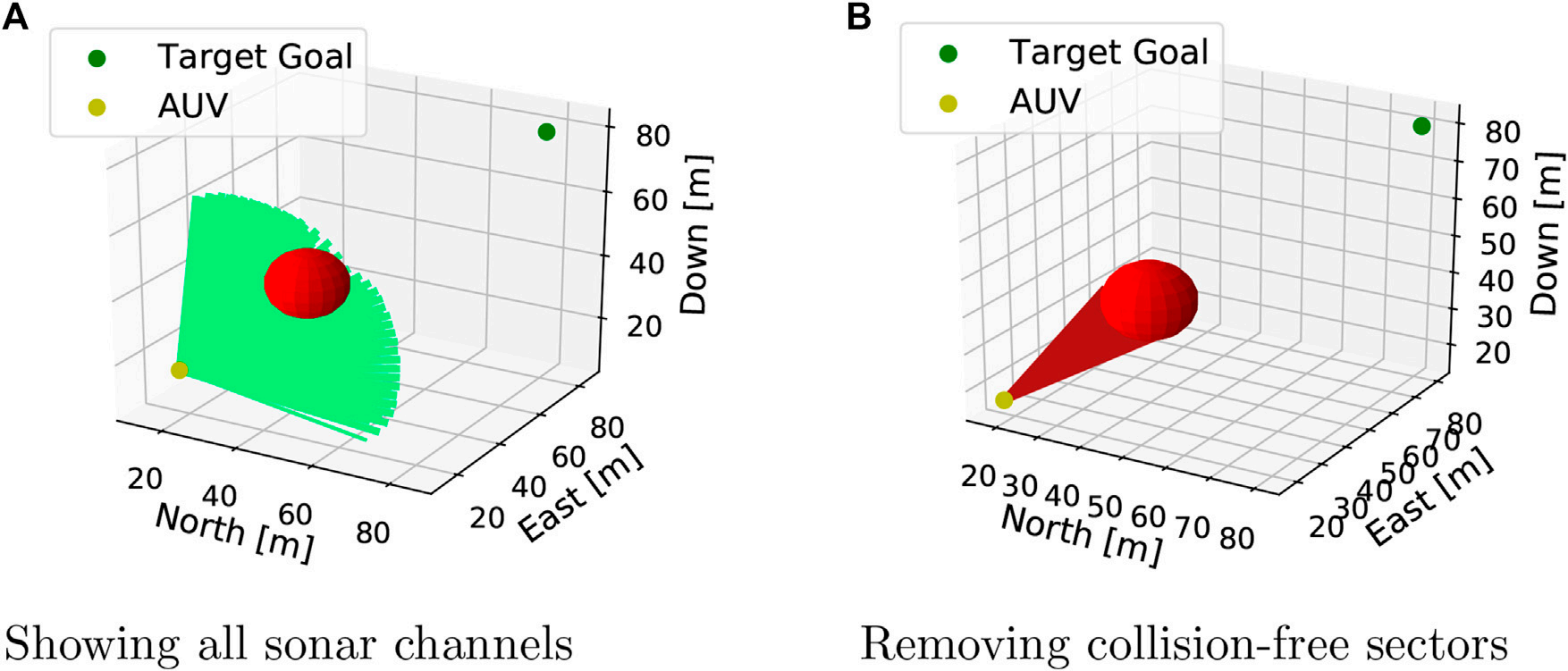
Rendering of the sonar simulation during an active episode.

Being able to react to the unforeseen obstacles requires the AUV to perceive the environment through sensory inputs. This perception, or obstacle detection, is simulated by providing the agent a 2D sonar image, representing distance measurements to a potential intersecting object in front of the AUV. This setup emulates a Forward Looking Sonar (FLS) mounted on the front of the AUV. A 3D rendering of the FLS simulation is seen in Figure 2. The specific sensor suite, the sonar range, and the sonar apex angle are configurable and can therefore be thought of as hyperparameters.

Depending on the sensor suite of choice, the number of sensor rays can get quite large. It is also notable that this issue is exponentially larger in 3D compared to 2D, slowing the simulation speed significantly as searching through the sonar rays (line search) is computationally expensive. For this reason, the sensor suite used in this research is 15 by 15, providing a grid with $10^{\circ }$ spacing between each sonar ray when scanning with a $140^{\circ }$ apex angle. This amounts to a total of 225 line searches per sensor update and in order to limit this stress on computational resources, the update frequency is set to 1Hz. Moreover, the sonar range is limited to 25m.

### 3.3 Reward Function

Reward function design is a crucial part of any RL process. The goal is to establish an incentive so the agent learns certain behavioral aspects. This is done by trying to imitate human-like behavior. For instance, following the path is objectively desirable, but this goal must be suspended in the case of a potential collision. When (and by what safety margin) to react is inherently a subjective choice. Regulating this trade-off is a balancing act, where following the path notoriously would result in many collisions and being too cautious would be ineffective. Additionally, a configuration involving excessive roll, i.e., the angular displacement of the AUV around its own longitudinal axis, is undesirable because that implies inverting or even swapping the two actuators’ effect (the rudder would operate as the elevator and vise versa) in terms of combating course and elevation errors. Not using the actuators too aggressively is therefore key to achieving smooth and safe operation. Thus, a reward function incorporating these important aspects of AUV motion control is developed.

The first part focuses on path following and simply penalizes errors between desired and actual course and elevation angle, as given by Equation 28:

$$r_{t}^{pf}\left(\tilde{\chi },\tilde{\upsilon }\right)=c_{\chi }{\tilde{\chi }}^{2}+c_{\upsilon }{\tilde{\upsilon }}^{2},$$ (28)

where $c_{\chi }$ and $c_{\upsilon }$ are negative weights deciding the severity of being off the course and elevation angles calculated by the guidance laws. The next incentive is avoiding obstacles blocking the path seen through the 2D sonar image. First, the range measurements are converted to a proportionally inverse quantity we have called *obstacle closeness*. This quantity is written as $c\left(d_{i,j}\right)=\text{clip}\left(1-\left(d_{i,j}/d_{\max}\right),0,1\right)$, where $d_{i,j}$ is the *i*’th and *j*’th pixel distance measurement and $d_{\max}$ is the sonar range. This transformation sets all sensor inputs zero as long as there are no obstacles nearby, effectively deactivating learning in this part of the neural net during the beginner scenario. The term incentivizing obstacle avoidance is written in Equation 29. It is calculated as a weighted average in order to remove the dependency on a specific sensor suite configuration. Furthermore, a small constant $\epsilon_{c}$ is used to remove singularities occurring when obstacle closeness in a sector is exactly one and $\gamma_{c}$ is a scaling parameter.

$$r_{t}^{oa}\left(d\right)=-\frac{\sum_{i\in ℐ}\sum_{j\in J}\beta_{oa}\left(\theta_{j},\psi_{i}\right){\left(\gamma_{c}\text{max}\left({\left(1-c\left(d_{i,j}\right)\right)}^{2},\epsilon_{c}\right)\right)}^{-1}}{\sum_{i\in ℐ}\sum_{j\in J}\beta_{oa}\left(\theta_{j},\psi_{i}\right)}.$$ (29)

**Figure 3:**
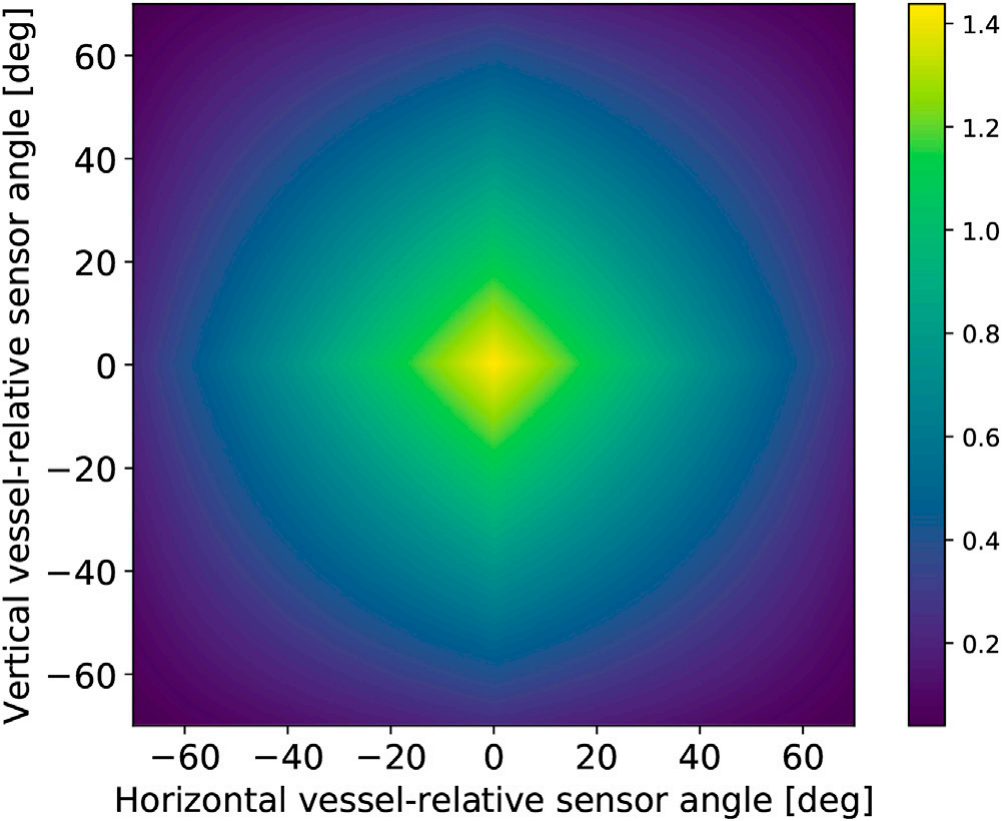
How the reward is scaled according to the sonar-data’s vessel-relative direction. Note that the grid illustrated is much finer than the 15 by 15 sensor suite used during simulation.

Since the vessel-relative orientation of an obstacle determines whether a collision is likely, the penalty related to a specific closeness measurement is scaled by an orientation factor dependent on the relative orientation. The vessel-relative scaling factor is written as $\beta_{oa}\left(\theta_{j},\psi_{i}\right)=\left(1-\left(2\left|\theta_{i}\right|/\gamma_{a}\right)\right)\left(1-\left(2\left|\psi_{j}\right|/\gamma_{a}\right)\right)+\epsilon_{oa}$. Here, $\epsilon_{oa}$ is a small design constant used to penalize obstacles at the edge of the configuration, and $\theta_{j}$ and $\psi_{j}$ define the vessel-relative sonar direction. Figure 3 illustrates how the 2D sonar image is weighted in terms of the sector importance given by $\beta_{oa}$. As is clear, obstacles that appear centermost in the sonar image will yield the largest penalty.

To find the right balance between penalizing being off-track and avoiding obstacles—which are competing objectives—the weight parameter $\lambda_{r}\in \left[0,1\right]$ is used to regulate the trade-off. This structure is adapted from the work by Meyer et al. (2020a); Meyer et al. (2020b), which performed similar experiments in 2D. In addition, we add penalties to roll, roll rate, and the use of control actuation to form the complete reward function:

$$r_{t}\left(\tilde{\chi },\tilde{\upsilon },d,\phi ,r,\delta_{r},\delta_{s}\right)=\lambda_{r}r_{t}^{pf}\left(\tilde{\chi },\tilde{\upsilon }\right)+\left(1-\lambda_{r}\right)r_{t}^{oa}\left(d\right)+c_{\phi }\phi^{2}+c_{r}r^{2}+c_{\delta_{r}}\delta_{r}^{2}+c_{\delta_{s}}\delta_{s}^{2}.$$ (30)

### 3.4 Feedback/Observations

The list of state observations, referring to the states of the dynamical model, and the agents' inputs during training and in operation is seen in Table 4. The inputs are normalized by the true or the empirical maximum, so that values passed into the neural network are in the range [−1,1]. Input normalization is used to improve the speed of convergence and the symbols are denoted by subscript *o* to indicate that these are the actual values passed as observations. The nonlinear activation functions of neural networks tend to saturate if the inputs get too large; hence, normalization is a means used to counteract this effect. Furthermore, large input values might lead to huge error gradients, which in turn causes unstable training. Normalization is therefore a simple form of preprocessing contributing to faster and more stable training (Yann LeCun et al., 1998).

**TABLE 4:** Observation table for end-to-end training for path following. All states and errors are normalized by the empirical or true maximum value.

Observation		Max
Relative surge + speed	$u_{ro}=\frac{u_{r}}{u_{\max}}\in \left[-1,1\right]$	2
Relative sway speed	$v_{ro}=\frac{v_{r}}{v_{\max}}\in \left[-1,1\right]$	0.3
Relative heave speed	$w_{ro}=\frac{w_{r}}{w_{\max}}\in \left[-1,1\right]$	0.3
Roll	$\phi_{o}=\frac{\phi }{\phi_{\max}}\in \left[-1,1\right]$	π
Pitch	$\theta_{o}=\frac{\theta }{\theta_{max}}\in \left[-1,1\right]$	π
Yaw	$\psi_{o}=\frac{\psi }{\psi_{\max}}\in \left[-1,1\right]$	π
Roll rate	$p_{o}=\frac{p}{p_{\max}}\in \left[-1,1\right]$	1.2
Pitch rate	$q_{o}=\frac{q}{q_{\max}}\in \left[-1,1\right]$	0.4
Yaw rate	$r_{o}=\frac{r}{r_{\max}}\in \left[-1,1\right]$	0.4
Course error	${\tilde{\chi }}_{o}=\frac{\chi_{d}-\chi }{\chi_{\max}}\in \left[-1,1\right]$	π
Elevation error	${\tilde{\upsilon }}_{o}=\frac{\upsilon_{d}-\upsilon }{\upsilon_{\max}}\in \left[-1,1\right]$	π
Ocean current velocity, surge	$u_{c,o}=\frac{u_{c}}{V_{c,\max}}\in \left[-1,1\right]$	1
Ocean current velocity, sway	$v_{c,o}=\frac{v_{c}}{V_{c,\max}}\in \left[-1,1\right]$	1
Ocean current velocity, surge	$w_{c,o}=\frac{w_{c}}{V_{c,\max}}\in \left[-1,1\right]$	1

In addition to the state observations, the neural network inputs a flattened 2D sonar image measuring closeness. It is possible to pass the sonar image directly through the neural network, essentially learning to map raw sensor data to control action. By the fact that neural networks are capable of representing any continuous nonlinear function, this should be feasible in theory (Nielsen, 2015). However, as this requires a high-dimensional observation space, a larger neural network is needed to learn a control law. In turn, a larger neural net requires more data and more updates to converge, prolonging an already time-consuming process. To address this issue, dimensionality reduction is performed by max pooling the raw closeness image from (15,15) to (8,8). While max pooling tends to be more restrictive (a high closeness measurement indicates a small distance between the vehicle and an object in a vessel-relative channel), the extra dimension that 3D offers provides a viable path to pass the obstacles in most cases. Moreover, being restrictive favors safety and obstacle avoidance.

For the neural networks, we utilize the *MLP-Policy* (multilayer perceptron) provided by Stable Baselines which incorporates a fully connected, two hidden-layer neural networks with 64 neurons in each layer using hyperbolic tangents (tan⁡h) as the activation functions. The input size and the output size are decided by the observation space and the action space, respectively. As we pass 14 state observations plus the 64-pixel output from max pooling the raw sonar image, the total input vector is of size 78×1. The action space is naturally the rudder and elevator fin commands, meaning a 2×1 output vector. The value of the parameters used during the training is given in Table 5.

**TABLE 5:** Parameter table for training and simulation setup.

PPO	Description	Value
α	Learning rate	2.5e-4
γ	Discount rate	0.99
λ	GAE parameter	0.95
τ	Entropy bonus coefficient	0.001
*T*	Number of steps per policy updates	1,024
*K*	Number of epochs	4
*M*	Batch size	64
*N*	Number of parallel actors	4
Environment		
Δ	Look-ahead distance	3
$n_{w}$	Number of training path waypoints	7
$\gamma_{a}$	Sonar span apex angle	140
$s_{r}$	Sonar range	25
−	Sensor suite	(15, 15)
−	Sensor min. pool output	(8, 8)
−	Sensor update frequency	1
$\left[V_{\min},V_{\max}\right]$	Ocean current intensity limits	[0.5,1]
$d_{a}$	End-goal acceptance radius	1
$T_{f}$	Control fins time constant	0.2
Reward function		
$c_{\chi }$	Course error penalty coefficient	−1
$c_{\upsilon }$	Elevation error penalty coefficient	−1
$\gamma_{c}$	Obst. closen. penalty scaling	−12.5
$\epsilon_{c}$	Minimum obstacle penalty closeness	−5e−3
$\epsilon_{oa}$	Minimum vessel-relative scaling	−0.05
$c_{\phi }$	Roll penalty coefficient	−1
$c_{r}$	Roll rate penalty coefficient	−1
$c_{\delta_{r}}$	Rudder action penalty coefficient	−0.1
$c_{\delta_{s}}$	Elevator action penalty coefficient	−0.1
$\lambda_{r}$	path following/COLAV trade-off	[0.9,0.5,0.1]

## 4 Results and Discussions

This section covers the results obtained from applying the finalized DRL controllers in the various scenarios introduced in Section 3.1. Firstly, test reports from quantitative tests, which are obtained by running the simulation for a large sample of episodes and calculating statistical averages, are given. In light of these results, the behavioral aspects can be interpolated to visualize and pinpoint clearer trends. Secondly, the reports from testing the controllers in special-purpose scenarios are shown and analyzed to qualify if the agents have indeed learned to operate the AUV intelligently. Three values for the trade-off parameter $\lambda_{r}$ were used during the training to obtain three expert level controllers. This gives rise to a rational hypothesis on the test outcomes. The agent trained with $\lambda_{r}=0.9$ should on average yield a lower tracking error, while maintaining a higher collision rate. The reversed results should be seen in the case of $\lambda_{r}=0.1$.

### 4.1 Quantitative Results

**Figure 4:**
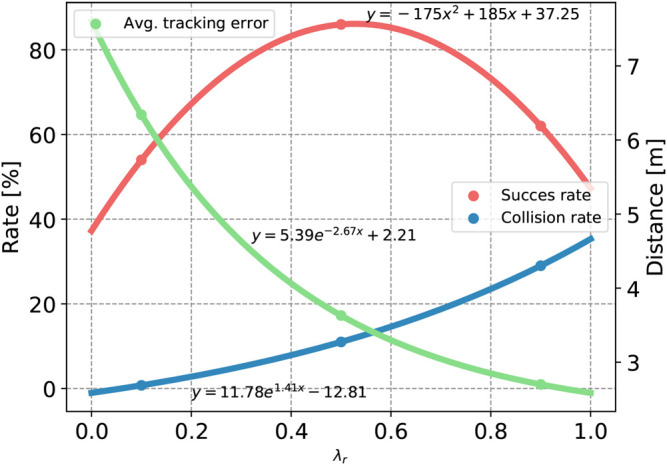
Curve-fitted data from Table 4. The average tracking error and the collision are fitted to exponential functions, while the success rate is fitted to a quadratic polynomial.

The quantitative results are obtained by running each training scenario, configured randomly in each episode, for N=100 episodes. As metrics, we use success rate, collision rate, and average tracking error over all episodes. Success is defined as the agent reaching the last waypoint within an acceptance radius of 1m from the destination without colliding. Equivalently, a collision is deemed to have happened if the distance between the AUV and any obstacle, at any time during an episode, is less than a specified safety radius $d_{\text{safety}}=1\text{m}$. Table 6 lists the full report from the quantitative tests. The results show a clear connection to the hypothesis that higher $\lambda_{r}$ should result in lower tracking errors but a higher collision rate on an average. Conversely, low $\lambda_{r}$ should result in fewer collisions but higher average tracking error. This matches exactly the expectation. The quantitative results can be interpolated to find general expressions for the success rate, collision rate, and average tracking error as functions of $\lambda_{r}$. The collision rate and the average tracking error are well described by exponential functions $y=ae^{bx}+c$. It is also seen that a quadratic function $y=ax^{2}+bx+c$ describes the success rate as a function of the trade-off parameter quite well. This matches the expectations as higher $\lambda_{r}$ induces more collisions and therefore lowers the success rate. On the other hand, during the episodes where the agent manages to avoid collisions, it always succeeds because the tracking error is very low. Lower $\lambda_{r}$ configurations naturally have the opposite problem. The low collision rate is due to being more willing to go off track but makes it less likely to reach the end goal within the acceptance radius. Figure 4 plots the data points from Table 4 together with the curve-fitted functions of $\lambda_{r}$.

**TABLE 6:** Test results from sampling N=100 random training scenarios.

Trade-off	Metric	Intermediate	Proficient	Advanced	Expert	Avg.
	Success rate [%]	68	66	62	52	62
$\lambda_{r}=0.9$	Collision rate [%]	16	28	34	38	29
	Avg. tracking error	1.67	2.91	3.14	3.09	2.70
	Success rate [%]	100	100	86	59	86
$\lambda_{r}=0.5$	Collision rate [%]	0.00	0.00	8.00	36.0	11
	Avg. tracking error [m]	1.97	3.76	4.44	4.33	3.63
	Success rate [%]	65	68	45	54	54
$\lambda_{r}=0.1$	Collision rate [%]	0	0	0	3	0.75
	Avg. tracking error [m]	3.98	6.15	7.91	7.33	6.34

### 4.2 Qualitative Results

**Figure 5:**
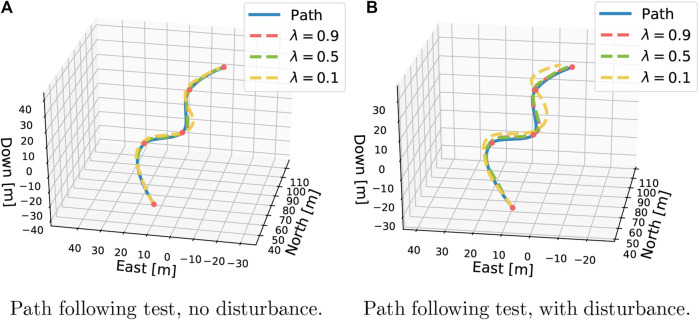
The pure path-following test. As expected, higher $\lambda_{r}$ are better at path following.

In the qualitative tests, four different scenarios (Section 3.1) are set up in order to test different behavioral aspects of the controllers. The first test sees the controllers tackle a pure path-following test, both with and without the presence of an ocean current. Figure 5 plots the results of simulating one episode.

For $\lambda_{r}=0.9$, an average tracking error of 0.45m and 0.52m in the ideal and disturbed environment is obtained, respectively. For $\lambda_{r}=0.5$, we obtained 0.54m and 0.98m. Finally, $\lambda_{r}=0.1$ achieved 1.64m and 3.95m. This amounts to a 15%,81%, and 141% increase in tracking error due to the disturbance, respectively. When testing, all controllers are run in deterministic mode to ensure that all results are reproducible. For the same reason, the current is fixed at a constant intensity and direction. From the test, we obtain the same performance observed in the quantitative tests. The agent tuned with $\lambda_{r}=0.9$ manages to obtain an average tracking error as low as 0.45m in ideal conditions, showcasing impressive tracking on curved 3D paths. Further, it is observed that the tracking errors increase significantly from $\lambda_{r}=0.5$ to 0.1. This is also reflected in the sensitivity of tracking error due to the presence of the disturbance. Most of the error happens where the path curvature is high. In addition, all cases are successful, except with the current disturbance, which is visibly off-track as it passes the last waypoint.

**Figure 6:**
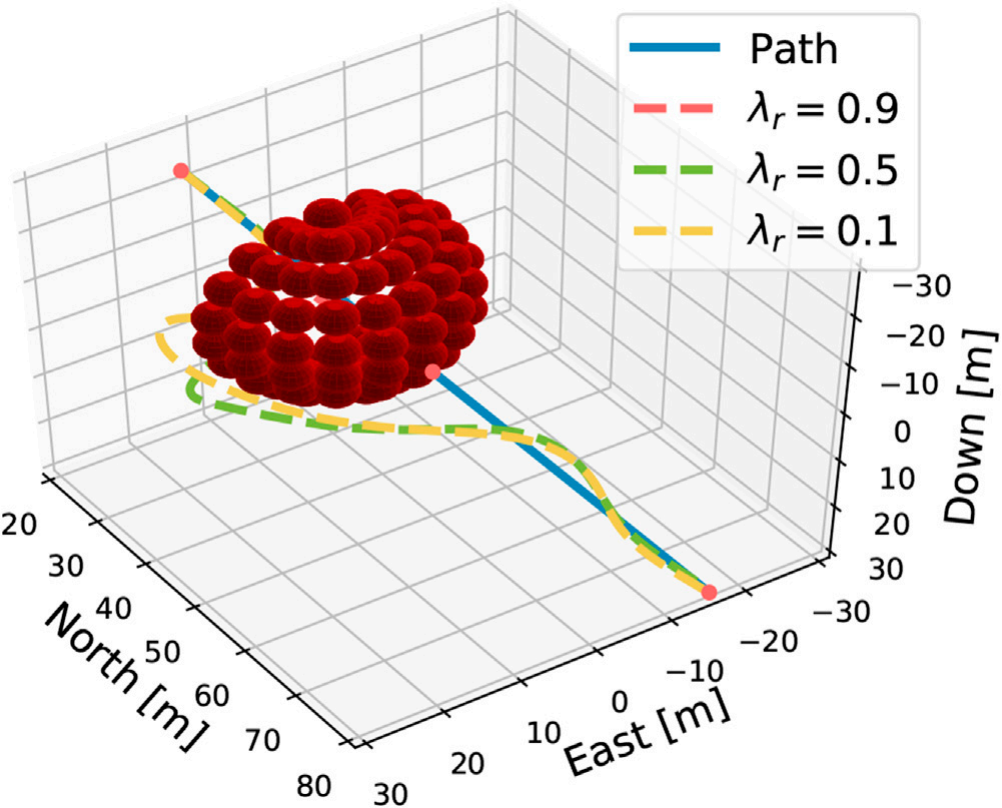
A dead-end test, where the obstacles are configuration as a half sphere with a radius of 20m.

A recurring problem seen when applying purely reactive algorithms is getting trapped in local minima, which in a practical sense materialize as dead ends. Therefore, the last test investigates if the agents have acquired the intelligence to solve a local minima trap in the form of a dead-end challenge. In addition, this can affirm the robustness and generality learned by the agents, as this is a completely novel situation. The obstacles are configured as a half sphere with radius 20 m. This means that the agent will sense the dead end 5m prior to the center (due to 25m sonar range) and must take the appropriate actions to escape it. The simulation, figured in Figure 6, shows that $\lambda_{r}=0.9$ fails in this test and cannot escape the dead end on account of it being too biased to staying on the path. On the other hand, $\lambda_{r}=0.5,0.1$ behaves somewhat similarly and manages to escape and reach the goal position. This is impressive performance as this scenario is novel for the agents and due to being a classical pitfall scenario for reactive algorithms.

**Figure 7:**
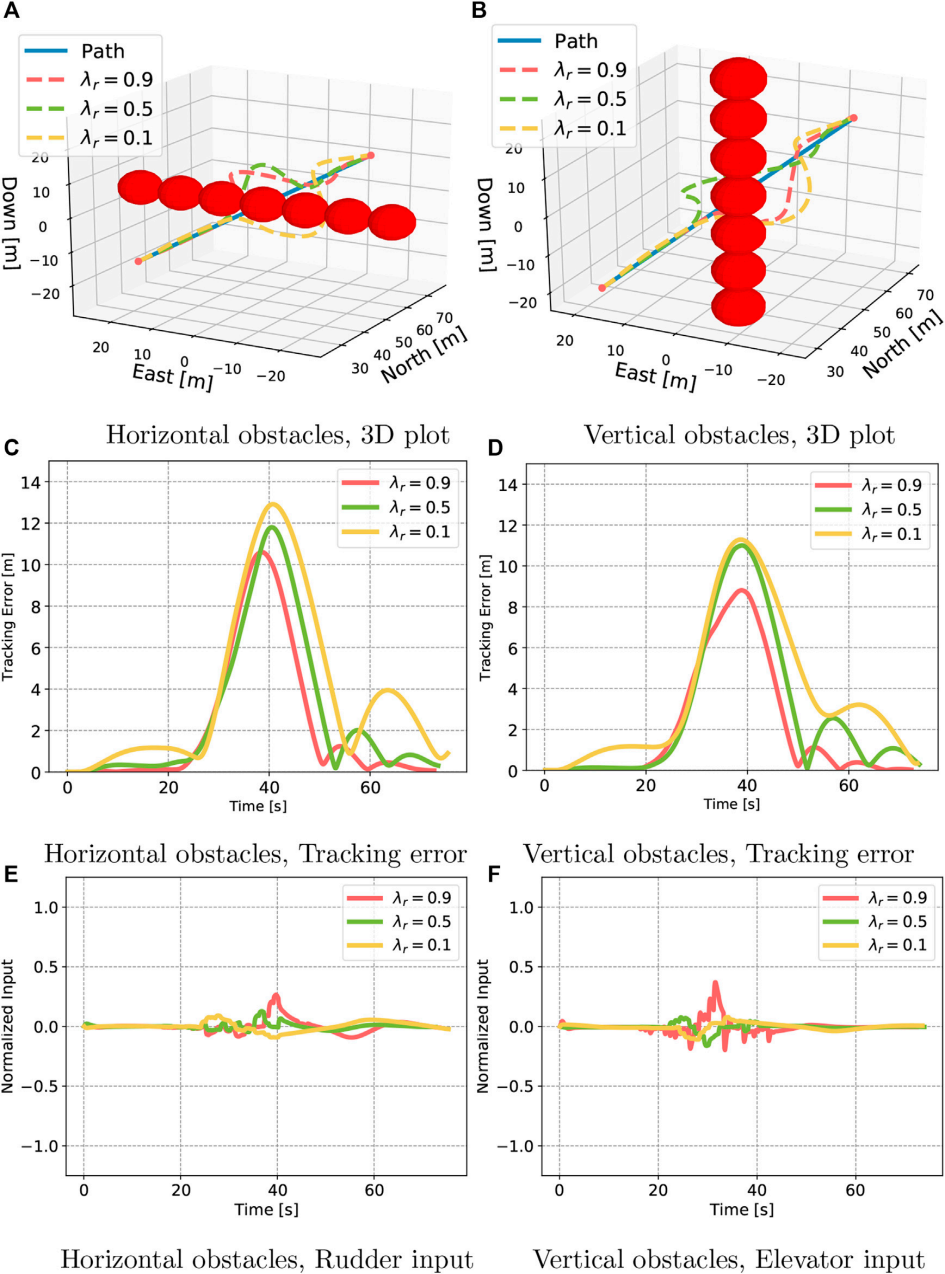
The horizontal and vertical obstacle test. Here, we are interested in seeing if the agent has learned which actuator to use to avoid the obstacles.

In the next test, we dissect if the agents learned to operate the actuators effectively according to how the obstacles are posed. In extreme cases, obstacles would be stacked horizontally and vertically, and optimally no control energy should be spent on taking the AUV towards “the long way around.” Instead, it should use the actuator in order to avoid the path on the lateral side of the stacking direction. Surely, an intelligent pilot would pass the obstacles in this manner. From the plots (Figure 7), it is observed that all agents waste little control energy using the “opposite” control fin. The agent with $\lambda_{r}=0.9$ uses more than the others due to being slower to react. It therefore has to spend more energy as it approaches the obstacle and might have to pull all levers to avoid collisions. The agent with $\lambda_{r}=0.1$ is seen to plan further ahead, as it takes action earlier than the other two, but it also travels far off the path. The controller with $\lambda_{r}=0.5$ can be seen to operate with human-like decision making. It steers clear of the obstacles in a nice and smooth curve, and it does not deviate in the plane that is not obstructed by obstacles.

The results obtained from the test scenarios demonstrate a clear connection to the reward function, as intended. In a pure path-following test, the agent biased towards path following manages to track the path with great precision. On the other hand, regulating the trade-off closer to COLAV yields agents that are willing to go further off track to find safe trajectories. This is reflected in the average tracking error and in the collision rate. Furthermore, it is seen that the latter controllers seem to react by spending less aggressive control. The controller tuned with $\lambda_{r}=0.5$ is seen to be effective in avoiding the obstacles and is also not deviating towards the suboptimal dimension. The expert level agent tuned with $\lambda_{r}=0.1$ shows great caution and from the quantitative analysis shows 99.25% collision-free samples out of 400, where collisions occurred at expert level difficulty only.

A current limitation in the simulated setup is the assumption that all states, including the ocean current, is available for feedback. We have therefore omitted the *navigation* part of the classical feedback loop for marine crafts. In a full-scale test, state estimation and sensor noise would naturally be part of the feedback loop, necessitating the need for a navigation module.

## 5 Conclusion and Future Work

In this research, DRL agents were trained using state-of-the-art RL algorithm PPO and deployed to tackle the hybrid objective of 3D path following and collision avoidance by an AUV. A *curriculum learning* approach was utilized to train the agent with increasing levels of complexities starting with path following, followed by the introduction of complexities in the obstacle layouts and ultimately the introduction of ocean currents. The AUV was operated by commanding three actuator signals in the form of propeller thrust, rudder, and elevator fin angles. A PI-controller maintained a desired cruise speed, while the DRL agent operated the control fins. The agent made decisions based on the observation of the state variables of the dynamical model, control errors, the disturbances, and sensory inputs from an FLS. The main conclusions are as follows:

• It was observed that agents biased towards path following achieved the objective with an average error of 0.5m even in the presence of a perturbing ocean current, clearly indicating its utility in the 3D case for vehicles with 6-DOF and multiple control fins.
• Quantitative evaluation was performed using statistical averages by sampling N=100 episodes per difficulty level and measuring the success rate (reaching the last waypoint within an acceptance radius without collision), collision rate, and average tracking error. By giving the agents the ability to perceive the environment through an FLS and providing the right incentives, it was observed that the agents biased towards COLAV demonstrated great obstacle avoidance capability under ideal conditions. The best agent accomplished zero collisions out of 300 samples without an ocean current and three out of 100 with the ocean current. The DRL controllers were also tested in special-purpose scenarios to investigate the quality of path following in the special cases where no objects are restricting the path, and optimal use of actuators in extreme obstacle configurations and in a dead-end test. Testing showed that the agents indeed had learned to maneuver the AUV effectively applying most control action in the unobstructed direction when encountering extreme obstacle configurations. Moreover, the agents with less incentive to stay on path managed to escape the local minima trap involved in the dead-end challenge. Hence, the results indicate that the agents had acquired enough general knowledge about the system, to make intelligent decisions when faced with novel situations.
• A reward system based on quadratic penalizations was designed to incentivize the agent to follow the path and also was willing to deviate if further on-path progress was unsafe. In addition, avoiding excessive roll and use of control actuation was avoided by penalizing such behavior. As path following and avoiding collisions are competing objectives, the agent must trade off one for the others in order to achieve a successful outcome in an episode. Since this trade-off is nontrivial, a regulating parameter $\lambda_{r}$ was introduced and tuned with three different values to observe behavioral outcome. Both the quantitative and qualitative evaluation confirmed the intended relationship between behavioral outcome and the trade-off regulation parameter. In addition, the training history revealed differences in adaptability and exploration/exploitation as the learning process advanced. The implications of this finding are that specific incentives make the agents more prone to certain weaknesses, which then should be addressed when setting up the learning process.

From the current studies, it is clear that DLR using curriculum learning can be an effective approach to taming an underactuated AUV with 6-DOF to achieve the combined objective of path following and collision avoidance in 3D. However, it is also important to stress that despite the demonstrated potential of the DRL approach holds, it will have very limited acceptability in safety-critical applications because the whole learning process happens in a black-box way, thereby lacking its explainability and analysability. Part of this black-box nature is attributed to the deep neural network that is at the heart of DRL because they lack functional expressibility. To address this issue, the learning of the trained agent can be put in the form of equations using symbolic regression. The symbolic regression based on gene expression programming has been demonstrated to discover new physics and equations directly from sparse data Vaddireddy et al. (2020). This will enable stability analysis of the system to make them more reliable but this kind of research is in its infancy at the moment.

## Data Availability Statement

The original contributions presented in the study are included in the article/Supplementary Material; further inquiries can be directed to the corresponding author.

## Author Contributions

SH developed the software framework facilitating this research and is the lead author. AR and OS supervised the research and provided guidance throughout the process, as well as proofreading.

## Conflict of Interest

AR is employed by SINTEF Digital.

The remaining authors declare that the research was conducted in the absence of any commercial or financial relationships that could be construed as a potential conflict of interest.

The reviewer ML declared a shared affiliation, with no collaboration, with the authors SH and AR to the handling editor at the time of the review.

## Footnotes

^1^ Link: https://github.com/simentha/gym-auv
